# Self-selected versus imposed running intensity and the acute effects on mood, cognition, and (a)periodic brain activity

**DOI:** 10.1007/s11571-024-10084-2

**Published:** 2024-03-01

**Authors:** Leonard Braunsmann, Finja Beermann, Heiko K. Strüder, Vera Abeln

**Affiliations:** 1 Institute of Movement and Neurosciences, German Sport University Cologne, Am Sportpark Muengersdorf 6, 50933 Cologne, Germany; 2 Albert-Ludwigs University of Freiburg, Freiburg, Germany

**Keywords:** Physical exercise, Autonomy, Self-determination, EEG, Neural noise, Mental health

## Abstract

The beneficial psychological effects of exercise might be explained by self-determination theory and autonomy. However, the underlying neurophysiological mechanisms are even less elucidated. Previously neglected, aperiodic (1/f) brain activity is suggested to indicate enhanced cortical inhibition when the slope is steeper. This is thought to be associated with an increased cognitive performance. Therefore, we hypothesize that running with a self-selected intensity and thus given autonomy leads to stronger neural inhibition accompanied by psychological improvements. Twenty-nine runners performed two 30-min runs. First, they chose their individual feel-good intensity (self-selected run; SR). After a 4-weeks washout, the same speed was blindly prescribed (imposed run; IR). Acute effects on mood (Feeling Scale, Felt Arousal Scale, MoodMeter®), cognition (d2-R, digit span test) and electrocortical activity (slope, offset, 1/f-corrected alpha and low beta band) were analyzed before and after the runs. Both runs had an equal physical workload and improved mood in the Felt Arousal Scale, but not in the Feeling Scale or MoodMeter®. Cognitive performance improved after both runs in the d2-R, while it remained stable in the digit span test after SR, but decreased after IR. After running, the aperiodic slope was steeper, and the offset was reduced. Alpha activity increased after SR only, while low beta activity decreased after both conditions. The aperiodic features partially correlated with mood and cognition. SR was not clearly superior regarding psychological effects. Reduced aperiodic brain activity indicates enhanced neural inhibition after both runs. The 1/f-corrected alpha band may emphasize a different neural processing between both runs.

**Supplementary Information:**

The online version contains supplementary material available at 10.1007/s11571-024-10084-2.

## Introduction

It is well-established that physical exercise has a positive impact on mental health, manifested, for instance, by acute improvements in affective and mood states (Berger and Motl 2000; Penedo and Dahn 2005; Reed and Ones 2006; Liao et al. 2015) and in cognitive performance (Lambourne and Tomporowski 2010; Chang et al. 2012; Basso and Suzuki 2017). The self-determination theory (SDT; Deci and Ryan 1985a, b) with its three basic psychological needs (i.e., competence, relatedness, and autonomy) provides one theoretical explanation for the effects on self-determination and the associated psychological improvements. In particular, perceived autonomy is considered as a key factor. Exercising driven by self-regulation and a more internal locus of control induces stronger improvements on affect (i.e., affective valence and activation) and mood compared to externally controlled and regulated behavior (Ekkekakis 2009). Importantly, psychological responses and cognition can influence each other, such that a positive affect is linked to an increase of attention (Kleinstäuber 2013; Niven 2013). Latter was found to be improved after physical activity as well (Hillman et al. 2003; Scudder et al. 2012). Moreover, other cognitive domains can be enhanced after exercising, such as executive function or memory performance (Chang et al. 2012). While there is still debate and inconsistency in the literature about the positive effects of exercise on affect, mood, and cognition, and the SDT has to be proven, shining light on the underlying mechanisms by investigating objective physiological responses might support the clarification of the SDT and exercise-related benefits on brain state and function. Previous studies using electroencephalography (EEG) reported, for instance, increased activity in the alpha band (e.g. 8–12 Hz) and decreased beta activity (e.g. 12–35 Hz; Schneider et al. 2009a; Vogt et al. 2010; Brümmer et al. 2011), what was initially associated with a state of decreased cortical activation (for a review, see Crabbe and Dishman 2004). However, looking at the body of literature to date, there are heterogeneous results (Crabbe and Dishman 2004; Gramkow et al. 2020). One suggestion is to divide the large range of frequencies within the beta band, and investigate, for instance, low beta (e.g. 12–20 Hz) to determine more accurate effects (Hosang et al. 2022). Another potentially more significant reason for the inconsistency may be the influence of a neural parameter that has been neglected so far: the aperiodic brain activity.

**Fig. 1:**
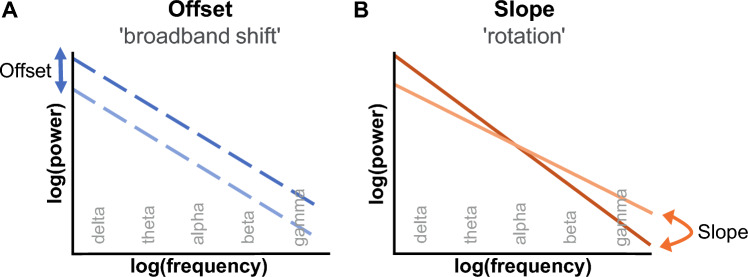
Schematic illustration of the aperiodic activity in a logarithmic power spectrum (based on Donoghue et al. 2020). **A**: Stimulus-induced changes in the spectral offset are manifested by a shift along the Y-axis. **B**: Stimulus-induced changes in the spectral slope are manifested by a rotation of the slope line (Podvalny et al. 2015) and may result in a shift of the offset (see Y-axis)

Electrocortical signals measured by EEG are not only composed of rhythmic, oscillatory patterns, that are investigated for about 100 years (Berger 1929), but also contain arrhythmic, aperiodic signals, which are present across all frequencies resulting in potential impacts on them (He 2014; Donoghue et al. 2020). This parameter, also called non-oscillatory activity, is characterized by a power-law form with a 1/f-like (‘one-over f’) distribution. This means that neural signals with a lower frequency have a high power and vice versa, representing the inverse relationship between power and frequency (Bak 1996; Buzsáki 2006). Graphically visualized in a power spectral density (PSD) plot by applying a double logarithmic scale on both axis (log power x log frequency), the relationship is represented by a linear line (Buzsáki and Draguhn 2004; see Fig. 1). The slope (α), one aperiodic feature, shows the steepness of the falloff based on the exponent β of 1/f^β^ (Buzsáki and Draguhn 2004; McSweeney et al. 2021) with β = − α (Lendner et al. 2020). Although the physiological origins of the 1/f brain activity have not been fully elucidated (Gao 2016), a widely discussed approach assumes that it reflects an excitation-inhibition (E:I) balance of neuronal activity (Gao et al. 2017; Chini et al. 2022). This concept assumes that homeostasis between synaptic excitation and inhibition is a prerequisite for efficient neuronal communication (Turrigiano and Nelson 2004; Vogels and Abbott 2009; Gao et al. 2017). Based on a computational neural circuit model, the E:I ratio is linked to the spectral exponent ß of the aperiodic activity (Gao et al. 2017). Therefore, this component might be useful to estimate the balance of E:I (Ahmad et al. 2022). For instance, an increased exponent accompanied by a steeper slope is considered to indicate a decreased E:I ratio. Furthermore, computational models found a link between the slope and the intensity of temporally correlated population spiking activity (Freeman and Zhai 2009; Pozzorini et al. 2013). Accordingly, a steeper (more negative) slope indicates lower background firing rates (Freeman and Zhai 2009) and more synchronized spiking activity, meaning that the neuronal population is highly correlated (Voytek et al. 2015). This neural adaptation is expressed, for instance, by performance improvements in cognitive and complex motor tasks (Ouyang et al. 2020; Immink et al. 2021). In contrast, a smaller spectral exponent, leading to a flatter slope gradient and thus a flatter spectrum, is associated with an increased E:I balance. This is thought to indicate higher background activation, meaning that neurons fire relatively asynchronously (Voytek and Knight 2015; Voytek et al. 2015). This is often interpretated as increased neuronal noise arising from neural decorrelation (Rubenstein and Merzenich 2003; Voytek and Knight 2015). A flatter slope is manifested, for instance, in a decreased working memory performance (Voytek et al. 2015; Thuwal et al. 2021). The offset, the second component of aperiodic brain activity, describes the neuronal activity independently of the frequency bands, which means across all frequencies (Donoghue et al. 2020; Numan et al. 2021). On the Y-axis of the log power spectrum, it marks the power of the lowest frequency under investigation (Numan et al. 2021). The offset responds to induced stimuli by a ‘broadband shift’ (Colombo et al. 2019; Merkin et al. 2021). Noticeably, the offset is considered less compared to the slope, but both features seem to be related to each other (Becker et al. 2018; Donoghue et al. 2020). In general, the 1/f activity has been found to vary with age (Voytek et al. 2015; Tran et al. 2020; McSweeney et al. 2021; Thuwal et al. 2021), seems to depend on the extent of sensory input during isolation (Weber et al. 2020), and correlates with various cognitive domains (González-Villar et al. 2017; Waschke et al. 2021).

How physical exercise affects the aperiodic brain activity has not been investigated to the best of our knowledge. However, as the 1/f activity is suggested to indicate neural excitability and cognitive performance, and exercise has been shown to impact these parameters, one could expect exercise-induced effects on the aperiodic component. Even though both aperiodic features, slope and offset, are calculated from the 1/f activity, implying an interaction with each other (Weber et al. 2020; Merkin et al. 2023), they still might reflect different physiological aspects (McSweeney et al. 2021). Therefore, the 1/f activity and a separate consideration of both aperiodic features might help to better understand the underlying neurophysiological mechanisms of psychological improvements following exercise.

The aim of the present study was to investigate how self-selected running versus imposed running acutely affects mood, cognition, and (a)periodic brain activity. As perceived autonomy is considered as a key factor according to SDT, we firstly assume that imposing the running speed hampers psychological outcomes. Accordingly, we hypothesize that the improvements in mood and cognition are more pronounced after the self-selected run compared to the imposed run. Regarding brain activity, we secondly expect that both runs lead to a decrease in both aperiodic features, as the spectral offset reflects mean neural population spiking activity and the spectral slope is associated with the neural E:I balance. This might be accompanied by a higher activity in the alpha band and lower activity in the low beta band. Comparing both runs, we speculate that the self-determination and autonomy during running augments electrocortical outcomes towards a stronger increase in alpha and decrease in low beta activity, a steeper slope, and a more reduced offset after running with a self-selected intensity compared to an imposed running intensity. As correlations between aperiodic activity and cognitive domains have previously been found, we intend to confirm this relationship and to expand this correlation by mood.

## Materials and methods

### Participants

Twenty-nine experienced recreational runners (14 females; mean age 22 ± 2.5 years) took part in this study (Table 1). The sample size was calculated using G*Power (v.3.1, Düsseldorf, Germany; Faul et al. 2007) indicating that 28 participants would allow to find significant changes in the behavioral parameters (i.e., attention, working memory, and mood) based on the power given in the literature (Reed and Ones 2006; Chang et al. 2012; McMorris and Hale 2012) in a within-subject design (Wilcoxon test: *d* = 0.5,* p* = 0.05). To take dropouts into account, we recruited a total of 30 participants. One participant had to be excluded due to an injury that occurred apart from the study. All subjects were non-smokers and right-handed, as assessed by self-report. No participant reported any health issues, including psychiatric or neurological conditions, or used any prescribed medication. Prior to any data collection, a written informed consent was obtained from each subject, and a detailed verbal and written explanation of the study was provided. In order to avoid influencing the subjects by knowing the aim of the study, the participants were only informed about the full purpose of the study at the end of all measurements. Subjects were compensated for their participation with 30 €. The study was conducted in accordance with the Declaration of Helsinki of 1964 approved by the ethic committee of the German Sport University Cologne (No. 040/2021).

**Table 1:** Participants characteristics

	Mean (n = 29)	Range	Male (n = 15)	Female (n = 14)
Age (years)	22.0 ± 2.5	19–28	23.0 ± 2.6	21.9 ± 2.3
Height (cm)	174.8 ± 9.4	158–190	181.7 ± 5.0	167.6 ± 7.4
Weight (kg)	70.7 ± 13.8	51–100	81.3 ± 10.0	59.4 ± 6.7
Body Mass Index (kg/m^2^)	22.9 ± 2.5	18.9–29.2	24.6 ± 2.3	21.1 ± 1.2
Running (times/week)	2.5 ± 0.8	1–5	2.2 ± 0.7	2.9 ± 0.8
Running (minutes/week)	112.4 ± 47.0	30–250	99.2 ± 43.9	126.6 ± 47.5

Values are displayed as mean ± SD

### Running trials

Starting in spring 2021, two 30-min runs were conducted on a 400-m outdoor running track. First, the subjects were asked to run at their individual feel-good intensity at a continuous pace, what is hereinafter referred to as a self-selected run (SR). Running speed and heart rate were blindly recorded. Four weeks later, the imposed run (IR) was performed, in which the identical running speed to the self-selected one was given. Importantly, the subjects were not aware that it was the same intensity. They were only asked to maintain the prescribed running speed, and if necessary, the pace was corrected by verbal instructions during the run. The aim was to ensure an identical running intensity during both runs while influencing the perceived autonomy by external instructions which is assumed, according to SDT, to reduce pleasure and motivation (Ekkekakis 2009; Vazou-Ekkekakis and Ekkekakis 2009). Since mainly moderate intensities have been reported to improve mood and cognition (Ekkekakis and Petruzzello 1999; Reed and Ones 2006; Chang et al. 2012; Erickson et al. 2019) and most individuals choose such an intensity when they select their own speed (Ekkekakis 2009), this was considered sufficient to achieve corresponding effects. The duration of 30 min was chosen as exercise-induced effects on mood (Reed and Ones 2006), cognition (Chang et al. 2012) and neurophysiological responses in the EEG (Woo et al. 2009) were strong or the strongest for this duration. To control for social effects and operation differences, the same experimenter performed the measurements with one subject at a time. Subjects ran alone to minimize social pressure, which otherwise could have led to increased speeds above the actual feel-good intensity (Berger and Motl 2000; Ekkekakis 2009). No other distractions during running, such as listening to music or similar, were allowed. Both runs were carried out at an interval of 4 weeks to take hormonal factors related to menstruation cycle of female participants into account. In addition, this time period was suggested to be long enough to serve as a washout phase to minimize carry-over effects of repeated measures, as randomization was not possible. On the other hand, 4 weeks were suggested to be short enough to minimize physical training-related effects. This is also why experienced subjects who trained on a regular basis were included. Furthermore, both runs were performed at the same time of day (± 9 min) to avoid circadian rhythm-related changes.

### Experimental measures

**Fig. 2:**
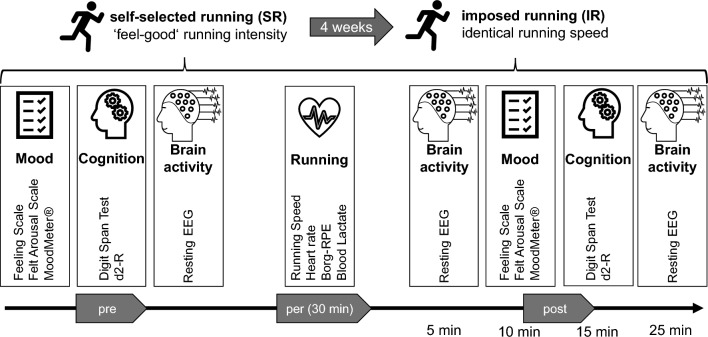
Experimental protocol. Participants were first asked to run for 30 min at their individual feel-good intensity. 4 weeks later, the same intensity was imposed. Before and after each run, measurements were performed on mood, cognition, and brain activity

The data were collected in a pre-post design as schematically shown in Fig. 2.

### Exercise data

During both runs, heart rate and running speed were permanently recorded using a Polar M400 running watch connected to a Polar H7 heart rate sensor (Polar Electro GmbH, Büttelborn, Germany) under blinded conditions. The display of the watch was taped off to avoid distraction or orientation while running. The experimenter had an additional watch (Garmin Forerunner 310XT; Garmin Ltd., Schaffhausen, Switzerland) connected to another heart rate sensor worn by the participants, to monitor and control the heart rate blindly for the participants during IR. To estimate the metabolic responses due to the exercise intensities, 20 µl of capillary blood were taken from the earlobe before running (pre), 1 min (post1), and 10 min after running (post10). Blood lactate concentrations (mmol/L) were analyzed after each test day in the laboratory using a BIOSEN C_line analyzer (EKF-Diagnostic, Barleben, Germany), and results were not communicated to the participants. The Rating of Perceived Exertion (RPE) scale according to Borg (1998) was used as a subjective measure and was assessed immediately after both runs. Additionally, a self-developed manipulation questionnaire with two questions was used once after IR, when all measurements were finished: (1) “How did you perceive the running intensity compared to the first run?” and (2) “How did you perceive the instructions of the running intensity?”. The subjects were asked to answer these questions on a seven-point Likert scale (1 = lower, 4 = equal, 7 = higher) and a five-point smiley analogue scale, respectively.

### Mood

Affect was assessed based on the circumplex model (Russell 1980; Ekkekakis and Petruzzello 1999) by means of the two single-item questionnaires Feeling Scale (FS; Hardy and Rejeski 1989) and Felt Arousal Scale (FAS; Svebak and Murgatroyd 1985), both as a paper-and-pencil version in German language with a good convergent validity (r = 0.50– to 0.73; Maibach et al. 2020). The FS measures affective valence on an eleven-point, bipolar scale (− 5 = very bad, 0 = neutral, + 5 = very good). The FAS assesses affective activation using a six-point, bipolar scale (1 = low, 6 = high). Additionally, the MoodMeter®, that was validated on a total of 645 people (Cronbach’s alpha interclass correlation coefficient 0.82 and 0.92; Kleinert 2006), was used to detect short-term changes of the perceived physical state (PEPS), psychological strain (PSYCH), and motivational state (MOT). It includes a short version of the "Eigenzustandsskala" (Nitsch 1976). It was presented as a paper-and-pencil version in German language containing 32-adjectives in mixed order for pre- and post- assessments, which had to be rated on a six-point Likert scale (0 = not at all, 5 = totally). All questionnaires were answered before (pre) and 11 min after both runs (post).

### Cognition

Two cognitive tests were carried out. The digit span test, a two-part subtest from the revised version of the Wechsler Memory Test with a test–retest reliability of 0.83 (Wechsler 2000) was used to test auditory attention and ultra-short-term memory (Lezak et al. 2004). In the forward digit span task, participants were first asked to immediately repeat verbally presented numbers with increasing sequence in the same order. Subsequently, the backward digit span task was performed, in which the participants repeated the numbers backwards, what additionally requires the manipulation of stored information to assess working memory. Sustained attention and concentration were measured using the d2-R as a paper–pencil test (Brickenkamp et al. 2010). This test consists of the letters ‘d’ and ‘p’ which are randomly arranged in 14 rows of 57 characters. Each one is marked with one to four small dashes either single or in pairs above or below the letter. Within 20 s per row, the task was to scan the lines and cross out as many d’s marked with two dashes while ignoring all other characters. The parameters analyzed were concentration performance (CP; number of crossed-out targets minus errors of commission), working speed (WS; sum of crossed-out targets), and working accuracy (WA; sum of all errors in relation to WS). Internal consistency (Cronbach's alpha) of the d2-R is high (CP: 0.92, WS: 0.91, WA: 0.90). Both tests were performed before (pre) and 15 min after both runs (post). Although a short-term training effect must be expected in attention tests, a systematic improvement during the 4 weeks can be excluded (Brickenkamp et al. 2010).

## Electroencephalography (EEG)

### EEG recordings

The EEG signals were recorded once before running (pre) as well as 5 min (post5) and 25 min (post25) afterwards. The data was continuously recorded for 5 min under resting state conditions, separated into 2.5 min with eyes open while fixing a point on the wall in front of the subjects, and subsequently 2.5 min with eyes closed. In order to prevent hemodynamic effects of changes in body position on cortical activity due to an acute shift of body fluids (Vanhatalo et al. 2003), the participants sat for 2 min before the start of the recording. During recording, the subjects remained seated in a relaxed position to prevent muscular contractions. Visual distractions were avoided, and noise was kept at a minimum. The electrode cap (EASYCAP GmbH, Woerthsee-Etterschlag, Germany) was mounted once, at the beginning of each session and was worn during the runs with the wires stored in a backpack to avoid irritations. An air-permeable cap was used to prevent an increase in heat during running. Markings were placed around the cap on the head to ensure identical position after running. We used Ag/AgCl active electrodes located at 32 scalp sites (Fp1, Fp2, F7, F3, Fz, F4, F8, FT9, FC5, FC1, FC2, FC6, FT10, T7, C3, Cz, C4, T8, CP5, CP1, CP2, CP6, TP10, P7, P3, Pz, P4, P8, TP9, O1, Oz, O2) plus one reference electrode (FCz) and one ground electrode (Fpz) based on the international 10–20 system (Jasper 1958). The number of electrodes were chosen to prevent inter-electrode cross talk due to sweat bridges after exercise (Reis et al. 2014). This has been proven to be a feasible procedure for exercise studies; for instance, other studies chose the number of electrodes of 30 (Ciria et al. 2018), 32 (Hicks et al. 2018), 64 (Spring et al. 2018) and other number of electrodes in between (for an overview see Gramkow et al. 2020). The cap was filled with a SuperVisc electrogel (EASYCAP GmbH, Woerthsee-Etterschlag, Germany) for optimal signal transduction. The signal was amplified using LiveAmp (BrainVision Inc., Morrisville, USA) with a sampling rate of 500 Hz.

### EEG preprocessing

Preprocessing and data analysis were carried out in MATLAB 2019a (The MathWorks Inc., Natick, Massachusetts, USA) using custom written code along with functions from the FieldTrip toolbox (Oostenveld et al. 2011). One subject had to be removed from the data analysis due to technical problems with the recording at IR only, resulting in n = 29 for SR and n = 28 for IR. After re-referencing the EEG data to the average across all channels, the raw data were bandpass filtered between 1 and 45 Hz using a 6th order Butterworth IIR filter in both forward and reverse directions. We excluded frequencies below 1 Hz to avoid potential slow artifacts due to sweating, what was expected after exercising. Based on visual inspection, bad channels were interpolated with the weighted neighbour approach (≤ 2 of channels per recording). Subsequently, the data was separated into the two segments of eyes open and eyes closed, and for both an independent component analysis (ICA) was applied. Components were visually inspected with respect to their topography and their time-series, and systematic artifacts such as eye blinks or cardiac artefacts were removed. A maximum of 2 out of 14 components were removed (eyes open: 1,2 ± 0,3; eyes closed: 0,6 ± 0,7). Next, we segmented the continuous recordings into non-overlapping epochs of 2 s. Subsequently, a semi-automatic z-transform-based artifact correction was performed with a z-value limit of 4. Trials exceeding this limit were excluded after visual inspection (eyes open: 14,2 ± 8,6; eyes closed: 12,2 ± 7,4). After that, we rejected trials if the variance of the signal exceeded the 1.5 times interquartile range of the median variance (Weber et al. 2020; eyes open: 0,5 ± 0,9; eyes closed: 0,8 ± 1,1). In a final step, we again visually inspected the signal for residual artifacts. At the end, we had a balanced number of trials for both conditions (SR eyes open 44,3 ± 8,8 and eyes closed 47,8 ± 9,6; IR eyes open 45,8 ± 10,9 and eyes closed 50,2 ± 10,2). Finally, data were reduced to 250 Hz for the further analysis.

### EEG analysis

**Fig. 3:**
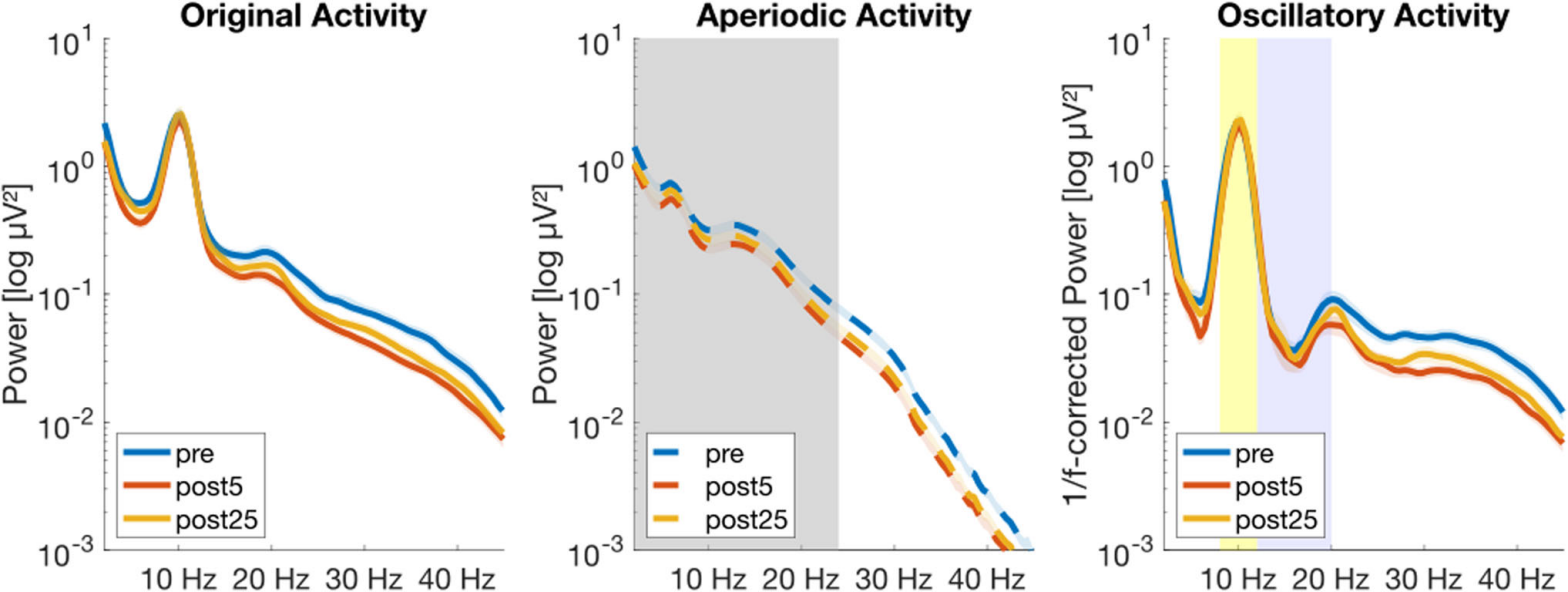
Power spectral density (PSD) plots showing exemplarily how the original EEG data was disentangled into the aperiodic and oscillatory components. A complete overview for all conditions can be found in the Supplementary Material (figure S1). PSD plots are presented as mean ± SEM in semi-log power space. **(Left)** PSD consisting of both, aperiodic and oscillatory components. **(Middle)** PSD after IRASA. The gray area marks the frequency range in which the aperiodic features were calculated (~ 1.9 to 23.7 Hz). **(Right)** PSD in which the aperiodic activity (1/f) was subtracted to obtain pure oscillations. The yellow area marks the alpha band (8–12 Hz), and the violet area marks the low beta band (12–20 Hz)

To decompose the brain activity into oscillatory and non-oscillatory components, we used Irregular-Resampling Auto-Spectral Analysis (IRASA; Wen and Liu 2016). Briefly, IRASA repeatedly resamples the EEG across a set of non-integer values *h* and their reciprocals 1/*h*. This up- and downsampling shifts any oscillatory peaks at higher and lower frequencies and thus attenuates any rhythmic component. The mean of each resampled spectra is then calculated before they are finally used to calculate the median. Thus, this process allows to isolate the 1/f (aperiodic) component of the data. For a full mathematical description of IRASA, see Wen and Liu (2016). We used default parameters for the decomposition algorithm (*h* = 1.1 to 1.9 in 0.05 steps), what allows an easier comparison to other studies that used the same parameters or described no deviating values (Wen and Liu 2016; Weber et al. 2020; Immink et al. 2021; Rosenblum et al. 2023). Additionally, the peak-widths in our PSDs (see Fig. 3) are not that large, thus higher resampling factors would not lead to even more peak-free aperiodic components (Gerster et al. 2022). Furthermore, it is recommended to keep *h*_max_ as small as possible (Gerster et al. 2022). Importantly, higher resampling values affect the evaluated frequencies, meaning that the effective frequency band range is reduced by a factor of 1.9 due to our maximal resampling factor (Wen and Liu 2016; Gerster et al. 2022). Accordingly, we examine the aperiodic results obtained within the frequency range of ~ 1.9 to 23.7 Hz. This fractal spectrum was used to calculate the key aperiodic features, slope and offset, by fitting a linear regression to the aperiodic signal in semilogarithmic power spectrum (polyfit.m, MATLAB and Curve Fitting Toolbox Release R2015a, The MathWorks Inc., Natick, Massachusetts, United States). Fitting was performed channel-wise on the trial-averaged data. Although Gao et al. (2017) originally analyzed the range between 30 and 50 Hz, subsequent studies used frequency ranges similar to ours to investigate aperiodic activity (e.g. Miskovic et al. 2018; Colombo et al. 2019; Rosenblum et al. 2023). Note that we use the term ‘steeper’ when the 1/f slope becomes more negative (higher exponent) and ‘flatter’ when the 1/f slope becomes more positive (lower exponent). To obtain the pure oscillatory brain activity unaffected by the fractal component, the aperiodic signal (1/f) was subtracted from the signal calculated with a regular spectral analysis. Using the 1/f-corrected oscillatory activity, we calculated frequency band analyses for alpha (8–12 Hz) and low beta activity (12–20 Hz). Based on current recommendations to better asses the effects of exercise on brain activity (Hosang et al. 2022), we decided not to investigate beta activity within a large range (e.g. 12–35 Hz), but to focus on the low beta band.

### Statistical analysis

Statistics were analyzed for exercise and behavioral data as well as control parameters using SPSS Statistics (Version 27; IBM, Armonk, New York, USA). The exercise data (running duration, speed, heart rate, and blood lactate values) of both runs were compared using paired t-tests, and the responses on the RPE scale were analyzed using Wilcoxon tests. For control parameters (temperature and the impact of COVID-19 on well-being), paired t-tests were calculated, and weather conditions were analyzed using Fisher’s exact tests. The manipulation questionnaire was evaluated descriptively based on the number of responses. For the behavioral data, mood (FS, FAS, MoodMeter®) and cognition (digit span test, d2-R), the acute changes after the runs were calculated by Wilcoxon tests or, in the case of missing requirements, by sign-tests. Pre and post scores of both runs for the behavioral data were compared using Wilcoxon tests or paired t-tests, respectively. Comparisons of the acute changes (Δ post–pre) were analyzed for both, mood and cognition, with paired t-tests. The statistical analysis of the EEG data was carried out in MATLAB 2019a (The MathWorks Inc., Natick, Massachusetts, USA) using custom written code along with functions from the FieldTrip toolbox (Oostenveld et al. 2011). We used non-parametric cluster-based permutation tests to test for changes over time in aperiodic slope and offset, as well as 1/f-corrected oscillatory activity. This approach provides insights into the spatial extent of the effect while still correcting for the multiple comparison problem using a non-parametric Monte Carlo randomization (Maris and Oostenveld 2007). In order to test the relationship between the aperiodic parameters slope and offset, Spearman correlation analyses were calculated, separately for pre, post5 and post25. To increase the statistical power, the EEG data for both runs (n_SR_ = 29 and n_IR_ = 28; note that we had to exclude one data set from IR due to technical reasons during recording) and their respective separation into eyes open and eyes closed conditions were combined [(29*2) + (28*2) = 114]. To test whether aperiodic features and the behavioral parameters were related to each other, we performed further correlation analyses. Therefore, we merged separately for the eyes open and eyes closed conditions, the pre and post EEG data of both runs (each n = 114) to pair them with the pre and post results of the respective mood or cognition parameter. We ensured that we used the post EEG measurements (post5 or post25) that were closest in time to the respective questionnaire or cognitive test (see Fig. 2). Thus, all mood questionnaires were combined with the post5 EEG data and all results from the cognitive tests were combined with the post25 EEG data. As the aperiodic pattern seems to appear more broadly spread across the scalp (He et al. 2010) and the understanding of the relationships to behavioral parameters is limited, we aim to clarify fundamental links between the aperiodic features and psychological measures. Accordingly, we calculated for all correlation analysis average slope and offset values across all electrodes for each participant. The correlation coefficients (*r*) ranges from − 1 to 1, in which the values of |0.1|, |0.3|, and |0.5| is considered as small, medium, and large effects, respectively, and the corresponding values for Cohen’s *d*_*z*_ are |0.2|, |0.5|, and |0.8| (Cohen 2013). The level of significance was set to *p* = 0.05.

## Results

### Exercise data

Running duration, speed, and intensity in terms of heart rate and blood lactate values did not differ between both runs (see Table 2). The RPE was higher after IR (14.5 ± 2.1), referring on average to a perceived exhaustion of ‘hard’ compared to ‘somewhat hard’ after SR (13.4 ± 1.5). The manipulation questionnaire, that was asked once at the end of all measurements, firstly revealed that 66% (n = 19) of the subjects perceived the intensity of IR to be higher than that at SR. Secondly, 76% (n = 22) of the participants stated on the smiley analogue scale that imposing the running speed was experienced as rather positive (n = 16) or positive (n = 6). The temperature was higher at IR (20.9 ± 4.1 °C) than at SR (12.9 ± 4.0 °C), while the weather conditions did not differ. The impact of COVID-19 on well-being on a scale from 0 to 10 was found to be lower at IR compared to SR.

**Table 2:** Comparison of exercise data

	SR (mean ± SD)		IR (mean ± SD)		Comparison SR versus IR		
					*p*	Effect size	
Duration (min)	30:26 ± 00:48		30:20 ± 00:42		.691	*d*_*z*_	0.075
Speed (km/h)	11.6 ± 1.4		11.6 ± 1,2		.870	*d*_*z*_	− 0.031
Heart rate (bpm)	162.5 ± 10.3		163.4 ± 9,0		.357	*d*_*z*_	− 0.181
% Heart rate max	85% ± 0.05%		85% ± 0.05%		.355	*d*_*z*_	− 0.181
Lactate pre (mmol/L)	1.2 ± 0.4		1.1 ± 0.3		.301	*d*_*z*_	0.196
Lactate post1 (mmol/L)	2.6 ± 1.8		2.7 ± 1.3		.614	*d*_*z*_	− 0.095
Lactate post10 (mmol/L)	1.5 ± 0.9		1.6 ± 0.7		.442	*d*_*z*_	− 0.145
Borg RPE (6–20)	13.4 ± 1.5		14.5 ± 2.1		.002*	*r*	0.573
Temperature (°C)	12.9 ± 4.0		20.9 ± 4.1		< .001*	*d*_*z*_	− 1.344
Weather (# measurements)	Sunny	4	Sunny	16	.457	*V*	0.247
	Cloudy	22	Cloudy	13			
	Rainy	3	Rainy	0			
Impact of COVID-19 (0–10)	4.0 ± 2.1		2.9 ± 1.6		.001*	*d*_*z*_	0.676

SR, self-selected run; IR, imposed run; SD, standard deviation; *p*, significance value; min, minute(s); km/h, kilometers per hour; max, maximum; bpm, beats per minute; mmol/L, millimol per liter (blood); RPE, rating of perceived exertion; °C, degree Celsius; #, number; *Significant (*p* < .05); d_z_, Cohen’s d in one-sample comparisons; r, correlation coefficient; V, Cramer’s V

### Mood

Activation in the FAS increased following both conditions (see Table 3). No differences between the runs were found for the pre scores, post scores or the acute changes (Δ post–pre). The runs did not lead to any changes in the FS. The pre scores and the acute changes did not differ between the runs, but the post scores were lower after IR than after SR. No acute changes in all MoodMeter® dimensions PEPS, PSYCH or MOT were found. The pre and post scores of PEPS and PSYCH were lower before and after IR.

**Table 3:** Results of the questionnaires for mood and cognitive tests

	SR (mean ± SD)				
	pre	post	∆	*p*	*r*
Feeling Scale	3.7 ± 0.7	3.9 ± 0.9	0.3 ± 0.8	.074	0.33
Felt Arousal Scale	3.4 ± 1.0	4.8 ± 1.0	1.4 ± 1.1	< .001*	0.79
MoodMeter® PEPS	3.9 ± 0.4	4.1 ± 0.5	0.2 ± 0.4	1.000	0
MoodMeter® PSYCH	3.9 ± 0.5	3.8 ± 0.5	− 0.2 ± 0.6	.070	− 0.34
MoodMeter® MOT	3.6 ± 0.4	3.7 ± 0.6	0.2 ± 0.5	.054	0.35
Digit span forwards	8.2 ± 1.9	8.3 ± 2.1	0.1 ± 1.7	.405	0.15
Digit span backwards	7.5 ± 1.8	7.7 ± 2.0	0.2 ± 1.6	.286	0.14
Digit span overall	15.7 ± 3.3	16.0 ± 3.7	0.3 ± 2.0	.678	0.08
d2-R WA	104.8 ± 8.1	108.6 ± 8.2	3.9 ± 6.7	.004*	0.55
d2-R WS	104.6 ± 10.5	115.8 ± 11.4	11.3 ± 5.3	< .001*	0.86
d2-R CP	104.2 ± 7.7	113.6 ± 9.3	9.4 ± 4.8	< .001*	0.87

	IR (mean ± SD)				
	pre	post	∆	*p*	*r*
Feeling Scale	3.3 ± 0.9	3.4 ± 1.1	0.1 ± 1.1	.479	0.13
Felt Arousal Scale	3.3 ± 1.0	4.8 ± 0.9	1.5 ± 1.0	< .001*	0.83
MoodMeter® PEPS	3.6 ± 0.7	3.8 ± 0.5	0.2 ± 0.7	.130	0.28
MoodMeter® PSYCH	3.6 ± 0.6	3.5 ± 0.5	− 0.1 ± 0.7	.580	− 0.10
MoodMeter® MOT	3.4 ± 0.7	3.5 ± 0.6	0.1 ± 0.7	.306	0.19
Digit span forwards	9.0 ± 1.7	8.2 ± 2.0	− 0.9 ± 1.9	.038*	− 0.39
Digit span backwards	7.8 ± 2.3	7.5 ± 1.8	− 0.2 ± 1.6	.263	− 0.10
Digit span overall	16.8 ± 3.4	15.7 ± 3.3	− 1.1 ± 2.4	.031*	− 0.42
d2-R WA	109.9 ± 8.7	112.5 ± 9.2	2.6 ± 5.7	.009*	0.53
d2-R WS	115.0 ± 10.5	120.8 ± 9.4	5.7 ± 4.5	< .001*	0.79
d2-R CP	113.5 ± 9.0	119.3 ± 9.1	5.8 ± 3.6	< .001*	0.84

	Comparison SR versus IR								
	pre			post			∆		
	*p*	Effect size		*p*	Effect size		*p*	Effect size	
Feeling Scale	.110	*r*	0.30	.016*	*r*	0.45	.493	*d*	0.13
Felt Arousal Scale	.686	*r*	0.08	.851	*r*	0.03	.602	*d*	− 0.10
MoodMeter® PEPS	< .001*	*r*	0.65	.004*	*r*	0.54	.717	*d*	− 0.07
MoodMeter® PSYCH	.002*	*r*	0.58	.005*	*r*	0.52	.698	*d*	− 0.07
MoodMeter® MOT	.051	*r*	0.36	.154	*r*	0.26	.699	*d*	0.07
Digit span forwards	.006*	*d*	− 0.55	.609	*d*	0.10	.029*	*d*	0.43
Digit span backwards	.343	*d*	0.18	.684	*d*	0.08	.332	*d*	0.18
Digit span overall	.006*	*d*	− 0.56	.535	*d*	0.12	.011*	*d*	0.51
d2-R WA	.007*	*d*	− 0.54	< .001*	*d*	0.72	.484	*d*	0.13
d2-R WS	< .001*	*d*	− 1.96	< .001*	*d*	− 0.83	< .001*	*d*	0.85
d2-R CP	< .001*	*d*	− 2.51	< .001*	*d*	− 1.35	< .001*	*d*	0.67

SR, self-selected run; IR, imposed run; SD, standard deviation; *p*, significance value; *d*_*z*_, Cohen’s d in one-sample comparisons; *r*, correlation coefficient; *Significant (*p* < .05); PEPS, physical state; PSYCH, psychological strain; MOT, motivational state; WA, working accuracy (sum of all errors in relation to WS); WS, working speed (WS; sum of crossed-out targets); CP, concentration performance (number of crossed-out targets minus errors of commission)

### Cognition

Cognitive performance (see Table 3) improved after both conditions in all dimensions of d2-R, naming working accuracy, working speed, and concentration performance. The comparisons of both interventions revealed that pre and post scores of all dimensions were higher before and after IR, and the acute changes were less pronounced for IR. The scores of the digit span test remained stable in all dimensions after SR, but after IR, the scores decreased in the forward version and in the overall score. In both dimensions, the participants showed higher scores before IR.

### Brain activity

The brain activity was disentangled into the aperiodic and oscillatory components (see Fig. 3). The values for the changes of the aperiodic features are provided in Table 4. The results of the post-hoc comparisons with the corresponding eyes open (EO) and eyes closed (EC) measurements are summarized in Table 5.

**Table 4:** Overview of the changes of the aperiodic features, slope and offset, across the measurements

	Running condition	Eyes	Measurement	Value (a.u.)	Δ to pre
Slope	Self-selected	Open	pre	− 1.88 (± 0.27)	
			post5	− 1.94 (± 0.29)	− 0,06
			post25	− 1.92 (± 0.31)	− 0,04
		Closed	pre	− 2.07 (± 0.22)	
			post5	− 2.12 (± 0.23)	− 0,05
			post25	− 2.12 (± 0.26)	− 0,05
	Imposed	Open	pre	− 1.93 (± 0.25)	
			post5	− 2.05 (± 0.24)	− 0,12
			post25	− 1.97 (± 0.28)	− 0,04
		Closed	pre	− 2.10 (± 0.19)	
			post5	− 2.22 (± 0.19)	− 0,12
			post25	− 2.19 (± 0.21)	− 0,09
Offset	Self-selected	Open	pre	1.11 (± 0.35)	
			post5	1.03 (± 0.33)	− 0.08
			post25	1.05 (± 0.36)	− 0.06
		Closed	pre	1.37 (± 0.35)	
			post5	1.29 (± 0.33)	− 0.08
			post25	1.34 (± 0.36)	− 0.03
	Imposed	Open	pre	1.16 (± 0.31)	
			post5	1.07 (± 0.31)	− 0.09
			post25	1.01 (± 0.32)	− 0.15
		Closed	pre	1.37 (± 0.28)	
			post5	1.31 (± 0.31)	− 0.06
			post25	1.32 (± 0.30)	− 0.05

### Aperiodic slope

**Fig. 4:**
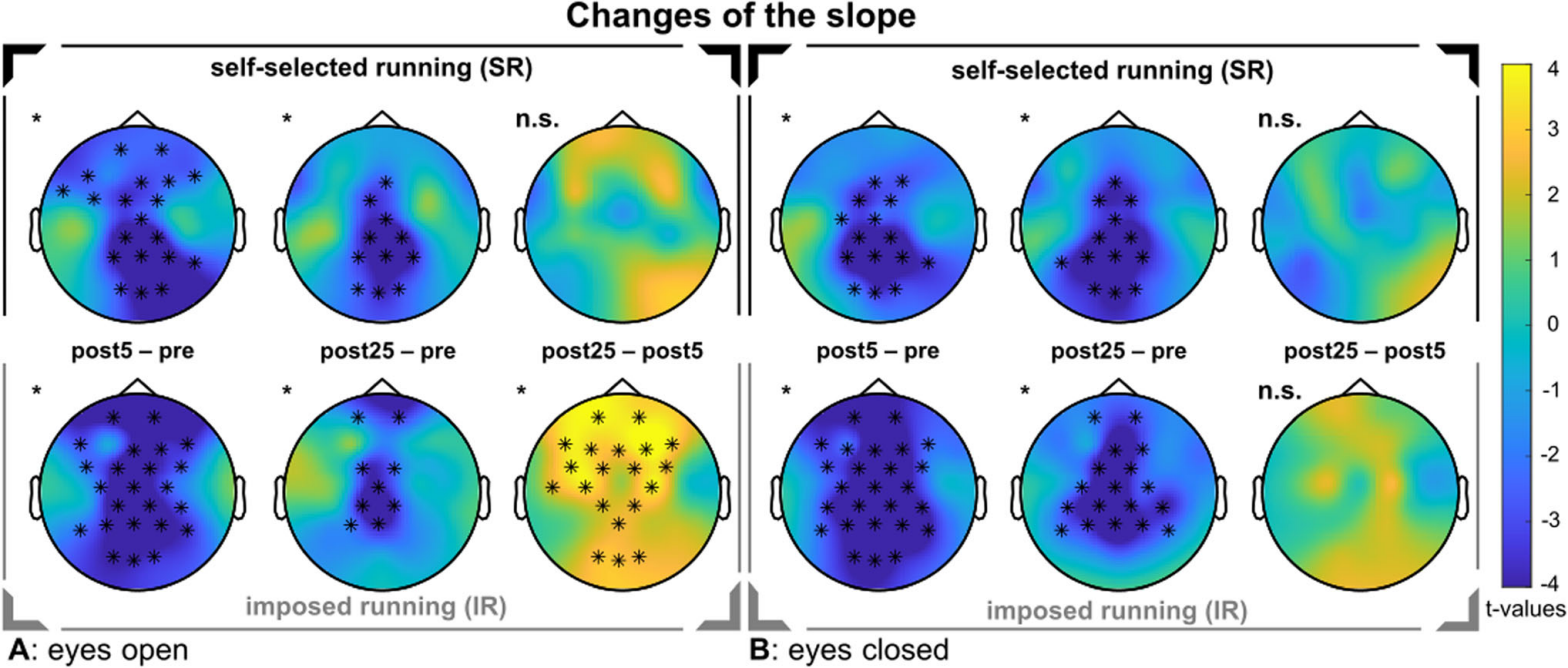
Changes in aperiodic slope. In the upper rows, the self-selected run is shown. In the bottom rows, the imposed run is shown. The topographical t-value distribution via cluster-based permutation tests using pairwise t-tests is plotted for the running-induced changes. In a gradient, the color blue indicates strongly negative t-values up to the color yellow, which represents strongly positive t-values. Significant electrodes within a cluster are indicated by 'x' for *p* < 0.05, and '*' for *p* < 0.01. **A** Changes in spectral slope in the eyes open condition. **B** Changes in spectral slope in the eyes closed condition

We observed a change in spectral slope over time for SR (EO: *sum*(*F*) = 180.50, *p* < 0.001; EC: *sum*(*F*) = 191.87, *p* < 0.001) and for IR (EO: *sum*(*F*) = 259.69, *p* < 0.001; EC: *sum*(*F*) = 339.28,* p* < 0.001). For both runs, follow-up pairwise comparisons revealed a decrease in spectral slope after 5 and 25 min, meaning that the slope became steeper, indicated by more negative values (see Fig. 4). Comparing both post measurements, IR showed an increase of the slope, but only in EO. The running-induced changes (∆5 min = post5–pre and ∆25 min = post25–pre) did not differ between SR and IR.

### Aperiodic offset

**Fig. 5:**
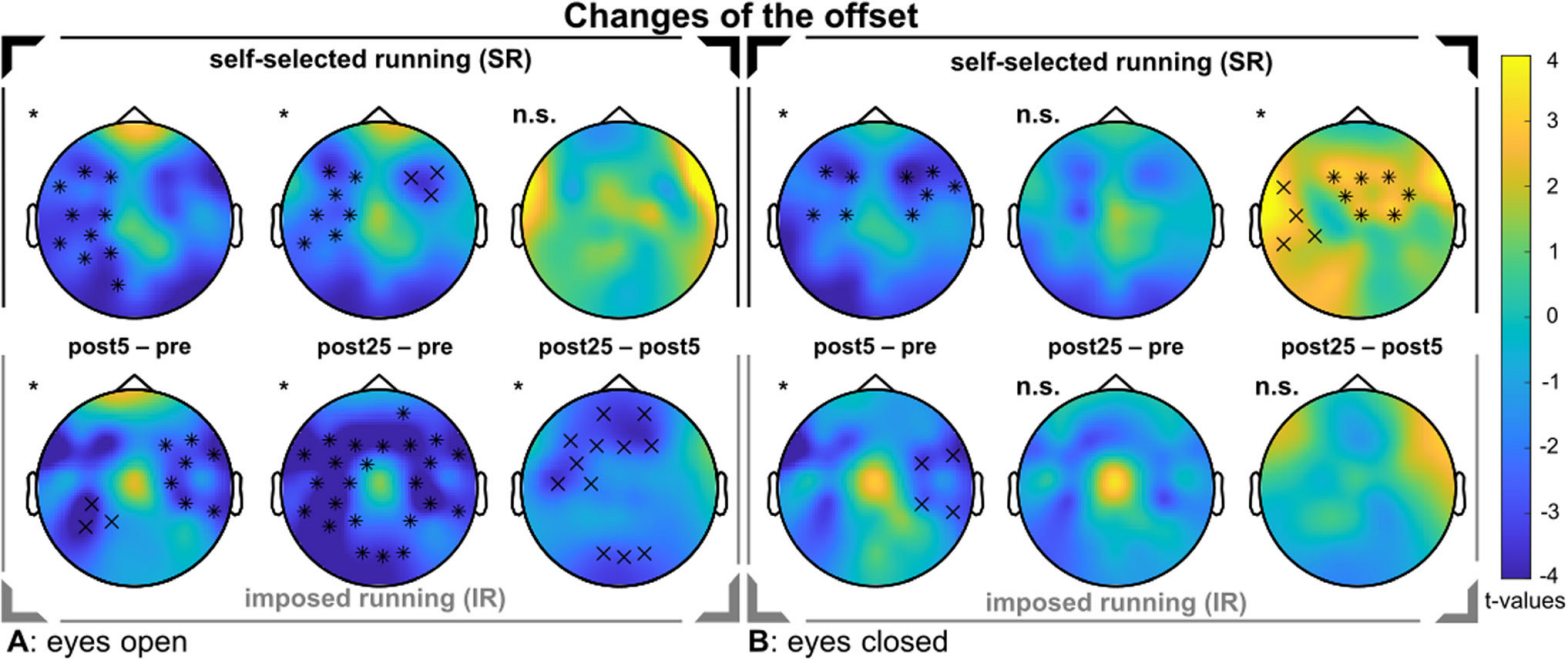
Changes in aperiodic offset. In the upper rows, the self-selected run is shown. In the bottom rows, the imposed run is shown. The topographical t-value distribution via cluster-based permutation tests using pairwise t-tests is plotted for the running-induced changes. In a gradient, the color blue indicates strongly negative t-values up to the color yellow, which represents strongly positive t-values. Significant electrodes within a cluster are indicated by 'x' for *p* < 0.05, and '*' for *p* < 0.01. **A** Changes in spectral offset in the eyes open condition. **B** Changes in spectral offset in the eyes closed condition

We observed a change in spectral offset over time for SR (EO: s*um*(*F*) = 67.01, *p* < 0.001; EC: *sum*(*F*) = 34.07, *p* = 0.006) and for IR (EO: *sum*(*F*) = 255.14, *p* < 0.001; EC: *sum*(*F*) = 25.42, *p* < 0.001). For both runs, follow-up pairwise comparisons revealed a decrease in the offset, meaning that the intercept on the y-axis was reduced after 5 and 25 min for EO, and for EC after 5 min only (see Fig. 5). Comparing both post measurements, we found for SR a re-increase in the EC condition and for IR a decrease in the EO condition. The running-induced changes (∆5 min = post5–pre and ∆25 min = post25–pre) did not differ between the runs.

### Correlation between slope and offset

Spearman correlation analyses revealed strong negative correlations between slope and offset for all three time points: pre (*r*_s_ = − 0.87, *p* < 0.001), post5 (*r*_s_ = − 0.80, *p* < 0.001), and post25 (*r*_s_ = − 0.81, *p* < 0.001).

### Oscillatory activity

**Fig. 6:**
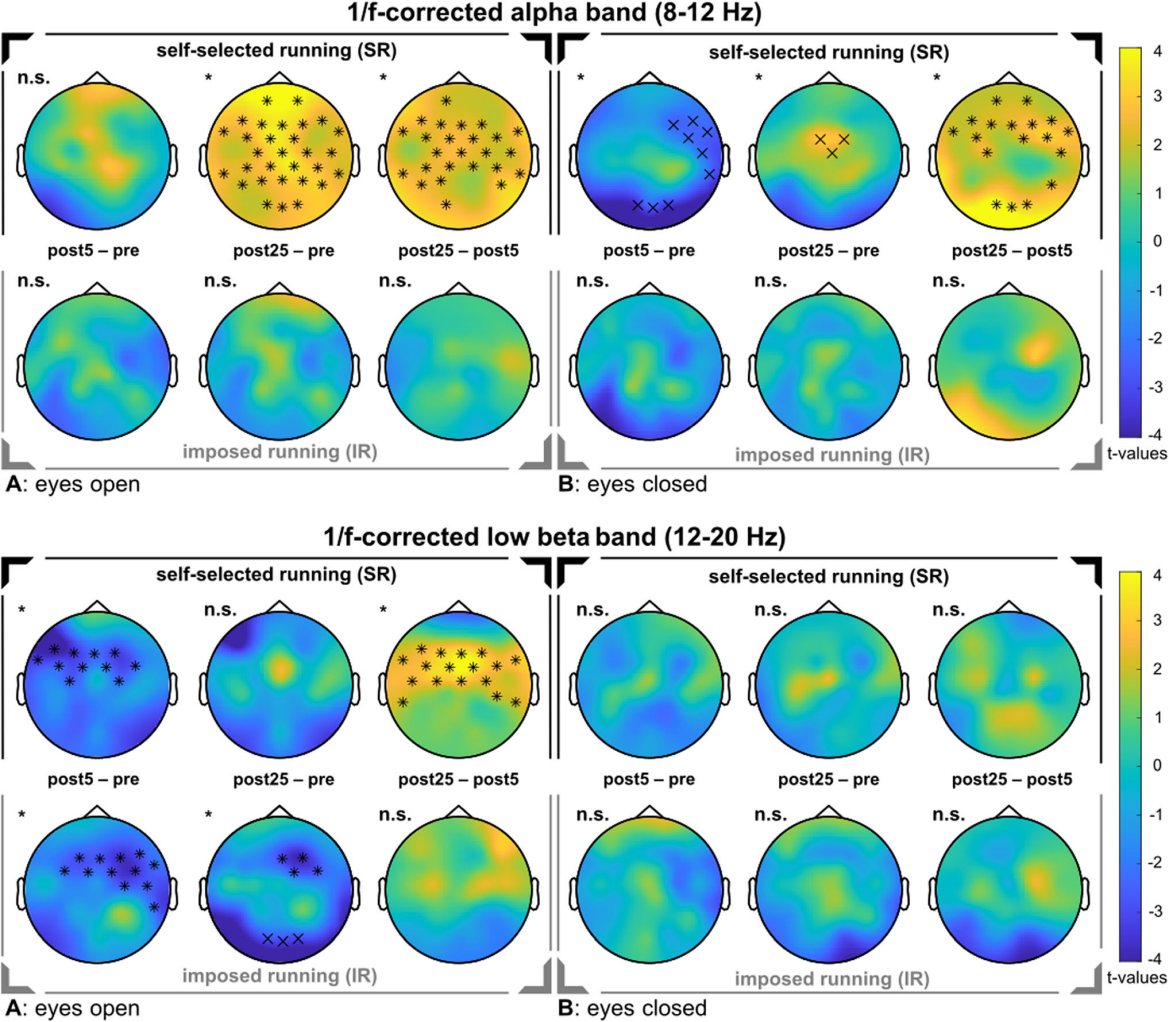
Changes in 1/f-corrected oscillatory activity in the alpha and low beta band. In the upper rows, the self-selected run is shown. In the bottom rows, the imposed run is shown. The topographical t-value distribution via cluster-based permutation tests using pairwise t-tests is plotted for the running-induced changes. In a gradient, the color blue indicates strongly negative t-values up to the color yellow, which represents strongly positive t-values. Significant electrodes within a cluster are indicated by 'x' for *p* < 0.05, and '*' for *p* < 0.01. **A** Changes in the eyes open condition. **B** Changes in the eyes closed condition

For the 1/f-corrected alpha band activity (8–12 Hz), we observed a change over time only for SR (EO: *sum*(*F*) = 184.68, *p* = 0.002; EC: *sum*(*F*) = 22.39, *p* = 0.019), but not for IR. Pairwise comparisons revealed a decrease in alpha activity 5 min after SR, but only with EC. After 25 min, the activity was increased in both, with EO and EC (see Fig. 6). Accordingly, we found an increased activity when comparing both post measurements.

For the 1/f-corrected low beta band (12–20 Hz), we observed a change over time after SR (EO: s*um*(*F*) = 77.87, *p* < 0.001) and after IR (EO: *sum*(*F*) = 71.92, *p* < 0.001), but both times only with EO. For the EO conditions, follow-up pairwise comparisons revealed a decrease in beta activity 5 min after SR, but not after 25 min. Accordingly, a re-increase of low beta activity was shown when comparing both post measurements. For IR, follow-up pairwise comparisons revealed a decrease in beta activity after 5 and 25 min (see Fig. 6). The comparisons of the differences between both interventions (∆5 min = post5–pre and ∆25 min = post25–pre) revealed no differences for either alpha or low beta activity (Table 5).

**Table 5:** Post-hoc comparisons for the aperiodic features, slope and offset, and the 1/f-corrected alpha and 1/f-corrected low beta bands

	Running condition	Eyes	Comparison	sum(*t*)	*p*	Significant electrodes	Single-subject level
Slope	Self-selected	Open	post5–pre	− 70.24	.001	20	65.5%
			post25–pre	− 40.85	.002	11	72.4%
			post25–post5	No cluster	n.s	–	–
		Closed	post5–pre	− 54.69	.001	15	75.9%
			post25–pre	− 53.35	.002	13	79.3%
			post25–post5	No cluster	n.s	–	–
	Imposed	Open	post5–pre	− 100.26	.001	24	85.7%
			post25–pre	− 30.92	.002	9	71.4%
			post25–post5	62.72	.001	20	75.0%
		Closed	post5–pre	− 117.33	.001	26	89.3%
			post25–pre	− 72.13	.001	18	82.1%
			post25–post5	No cluster	n.s	–	–
Offset	Self-selected	Open	post5–pre	− 30.59	.002	20	69.0%
			post25–pre	− 17.54	.002	13	62.1%
			post25–post5	No cluster	n.s	–	–
		Closed	post5–pre	− 15.83	.007	13	75.9%
			post25–pre	No cluster	n.s	–	–
			post25–post5	18.47	.006	15	69.0%
	Imposed	Open	post5–pre	− 21.60	.002	13	67.9%
			post25–pre	− 94.31	.001	24	92.9%
			post25–post5	− 26.62	.012	15	67.9%
		Closed	post5–pre	− 12.49	.001	4	60.7%
			post25–pre	No cluster	n.s	–	–
			post25–post5	No cluster	n.s	–	–
1/f–corrected alpha activity	Self-selected	Open	post5–pre	No cluster	n.s	–	–
			post25–pre	86.54	.001	30	72.4%
			post25–post5	60.71	.005	25	72.4%
		Closed	post5–pre	− 13.53	.014	12	72.4%
			post25–pre	7.93	.034	3	58.6%
			post25–post5	45.07	.004	18	69.0%
	Imposed	Open	post5–pre	No main effect over time			
			post25–pre				
			post25–post5				
		Closed	post5–pre				
			post25–pre				
			post25–post5				
1/f-corrected low beta activity	Self-selected	Open	post5–pre	− 32.55	.001	11	89.7%
			post25–pre	No cluster	n.s	–	–
			post25–post5	48.29	.001	17	72.4%
		Closed	post5–pre	No main effect over time			
			post25–pre				
			post25–post5				
	Imposed	Open	post5–pre	− 33.66	.001	12	89.3%
			post25–pre	− 12.63	.007	10	82.1%
			post25–post5	No cluster	n.s	–	–
		Closed	post5–pre	No main effect over time			
			post25–pre				
			post25–post5				

All results are corrected for multiple comparisons. The column “Single-subject level” represents the percentage of subjects who showed the indicated change (e.g. reduced slope) averaged across all electrodes. *p*, significance value; n.s., not significant

### Correlations between aperiodic brain activity and psychological parameters

We found a small negative correlation between the aperiodic slope and affective activation via FAS (EO: *r* = − 0.24, *p* = 0.011; EC: *r* = − 0.20, *p* = 0.037). Regarding cognition, the slope correlated negatively and to a small extent with working accuracy of the d2-R (*r* = − 0.22, *p* = 0.022; EC: *r* = − 0.21, *p* = 0.025) and the overall score of the digit span test (only with EO: *r* = − 0.21, *p* = 0.028).

Furthermore, we observed a small negative correlation between the aperiodic offset and the PEPS dimension of the MoodMeter® (EO:* r* = − 0.23, *p* = 0.012). Regarding cognition, the offset correlated negatively and to a small extent with working speed of the d2-R (EO:* r* = − 0.26, *p* = 0.005) and positively with the forward score (EO:* r* = 0.22, *p* = 0.019) and the overall score (EO:* r* = 0.21, *p* = 0.024) of the digit span test. Note that all correlations for the offset were only significant in the EO condition. An overview about all results can be found in the Supplementary Material (Table S2).

## Discussion

The study aimed to investigate acute effects of self-selected running (SR) in comparison to imposed running (IR) on psychological and neurophysiological parameters. We demonstrated that mood and cognition were partially improved after both runs, meaning that SR was not found to be clearly superior for psychological improvements. Aperiodic (1/f) brain activity revealed that the slope was steeper, and the offset was reduced after both runs. Furthermore, the aperiodic features slightly correlated with selected dimensions of cognition (e.g. working accuracy and speed) and mood (e.g. affective activation). Interestingly, the 1/f-corrected oscillatory activity seem to differ depending on the two running conditions as the alpha activity increased after SR only, whereas a decreased low beta activity was apparent after both runs.

### Exercise data

In first instance, it is important to notice, that both runs were equal in terms of running speed, duration, heart rate, and blood lactate concentrations, thereby ensuring comparability of the runs. The running intensity of approximately 85% HRmax (Tanaka et al. 2001) corresponds to other studies that examined self-selected intensities (Nabetani and Tokunaga 2001; Zamparo et al. 2001; Lind et al. 2008). The lactate values 1 min after each run (SR: 2.6 ± 1.8 mmol/L; IR: 2.7 ± 1.3 mmol/L) are consistent with Ekkekakis (2009), showing that most individuals choose a workload below up to close to their ventilatory or lactate threshold when exercising at self-chosen intensity (see also Lind et al. 2008; Parfitt et al. 2006). Note that the lactate sample collection 1 min after the end of the runs does not allow a valid classification of the metabolic processes. Instead, the lactate values were intended to assess the comparability of the runs. Therefore, based on these exercise parameters, we successfully imposed the same running bout as previously self-selected. RPE values indicate a perceived exertion after SR (13.4 ± 1.5) that was ‘somewhat hard’ (= 13), and thereby confirming other studies that have found similar values around 13 when a running intensity was self-chosen (Zamparo et al. 2001; Parfitt et al. 2006; Dias et al. 2014). However, it is remarkable that the perceived exertion after IR (14.5 ± 2.1) tended to be ‘hard’ (= 15), and thus was perceived higher, even though the same running speed was imposed. This was also reflected in the manipulation questionnaire, in which 2/3 of the subjects (66% or n = 19) perceived a higher intensity compared to SR, which contradicts the objective running parameters. The increased temperatures during IR (20.9 ± 4.1 °C) compared to SR (12.9 ± 4.0 °C) should be considered, though these were not unusual extreme temperatures, and the influence of the temperature does not seem to have a substantial effect on the RPE scale either (Sparks et al. 2005). After adjusting for temperature, analysis still revealed a difference in the Borg RPE between SR and IR (see Supplementary Table S1). Additionally, a potential influence of the COVID-19 situation on well-being can be neglected as the subjective ratings were generally low and even lower at IR.

### Mood

Both runs improved mood but only in terms of affective activation (FAS). This exercise-induced increase is consistent with the literature (Lind et al. 2008; Lattari et al. 2016). Lind et al. (2008) revealed differences in affective activation between a self-selected run and an imposed running speed. However, the imposed running speed was 10% higher than the self-selected one in their study. In contrast, in the present study, the exercise intensity of both runs was almost identical, which suggests that exercise intensity is of superior influence compared to autonomy. Furthermore, we did not observe any changes in affective valence (FS) or MoodMeter®. Only after adjusting for temperature, analysis revealed reduced scores in the FS after IR (see Supplementary Table S1). The lack of exercise-induced improvements and missing differences between both runs contradicts our assumption based on SDT with its Basis Psychological Needs Theory (Deci and Ryan 2000; Ryan and Deci 2002), suggesting that external regulation in exercise can deteriorate autonomy and consequently have negative effects on affective states (Ekkekakis 2009; Vazou-Ekkekakis and Ekkekakis 2009; Bartholomew et al. 2011). However, our results are in line with Schneider et al. (2009a), who did not observe changes in mood at low and preferred, but only at high exercise intensities, and there with deteriorating effects. Therefore, this might support the above-mentioned assumption that either the exercise intensity is more important than the perceived autonomy, or the intended impairments of imposing the running speed were not (strongly) perceived. In this regard, it is important to consider, that potential exercise-induced effects are depending on an individual autonomy versus control orientation based on previous experiences, as explained by Causal Orientation Theory (Deci and Ryan 1985b) of SDT. The importance of individual traits is also discussed in the exercise preference hypothesis, which assumes that exercise-induced changes in well-being depend on individual preferences, habituation effects, and previous exercise experiences (Schneider et al. 2009b; Brümmer et al. 2011). As our participants were athletes and sport science students, they were probably used to receive instructions, which is why they may not perceive them as a restriction of their autonomy. Instead, 76% (n = 22) of the participants stated that imposing the running speed was experienced as (rather) positive. This might explain that no clear differences between the runs in affect and mood were found. Additionally, a ceiling effect should also be considered, as the pre-values were already high in all questionnaires leaving not much room for improvements. Therefore, future studies should consider to test individuals who have less experience with exercise or populations that tend to have lower levels of well-being. Giving more instructions while running could also lead to a higher degree of autonomy restriction, which could result in more pronounced effects.

### Cognition

Sustained attention in the d2-R was improved after both runs. This is consistent with the literature that found enhanced cognitive performance (Lambourne and Tomporowski 2010; Chang et al. 2012; Basso and Suzuki 2017) and attention (Hillman et al. 2003; Scudder et al. 2012) after physical activity. Other studies that specifically used the d2(-R) also showed benefits following exercise interventions (Budde et al. 2008; Stroth et al. 2009; Kleppel 2016; Wollseiffen et al. 2016). These improvements could be attributed to an increased physiological arousal (Audiffren et al. 2008; Lambourne and Tomporowski 2010; McMorris and Hale 2012). This might be reflected psychologically as the results in the FAS confirm that affective activation was increased after both runs. Higher arousal might also explain the improved working speed of the d2-R after exercise, whereas accuracy increased to a smaller extent. Thus, our results support meta-analytic findings revealing that acute improvements in cognitive performance are primarily due to a faster working speed (McMorris and Hale 2012). The digit span test, the second cognitive test which did not rely on reaction time or speed, showed no acute changes in auditory attention and ultra-short-term memory after SR. Nevertheless, we found deteriorations after IR in the forward task and in the overall score, assuming that the IR was more prone to cause impairments. However, it is important to notice, that in both cognitive tests, the pre scores were higher before IR on the second assessment day than before SR. This might indicate long-term learning effects (Brickenkamp et al. 2010), although the study design intended to avoid carry-over effects by a 4-week period in-between runs. Furthermore, methodologically influencing factors were controlled as best as possible: The tests were always conducted by the same investigator and at the same time of day (Chang et al. 2012; Xu et al. 2021). Increased motivation before IR can also be excluded as subjects tended to report decreased motivation prior IR (*p* = 0.051) as assessed by the MoodMeter®. Due to the unequal pre running results, we avoid speculating about the effect between IR and SR here. Instead, our results confirm that running in general seems to have positive effects on cognitive performance.

### Brain activity

Five minutes after the end of both runs, the slope was reduced, i.e., it became more negative and thus the slope in the power spectrum was steeper. This effect proved to be stable after 25 minutes. The neurophysiological reduction of the slope indicates that the E:I balance decreased (Donoghue et al. 2020), meaning the inhibition of neuronal activity was greater than the excitation (Gao et al. 2017; Waschke et al. 2019; Chini et al. 2022). Interestingly, the results of the FAS showed increased psychological activation after both runs, clarified by the small negative correlation we found between slope and FAS. To the best of our knowledge, the current literature does not provide any findings regarding a relationship between aperiodic features and parameters of well-being. Even though the neural correlates are still not fully elucidated (King 2019), the present findings suggest that the aperiodic slope may be related to affective responses, but further studies are needed for clarification. Furthermore, the steeper slope might indicate lower background firing rates (Freeman and Zhai 2009) and more synchronized spiking activity (Voytek et al. 2015) leading to a reduction in neural noise (Pertermann et al. 2019). This was accompanied by improved cognitive performance after running, shown by the small negative correlation we found between spectral slope and accuracy in the d2-R. According to this, a more negative, steeper slope is associated with a higher level of accuracy. Furthermore, both aperiodic features correlated with the digit span test, meaning that a steeper slope and an increased offset correlated with higher performance in auditory attention and ultra-short-term memory. Additionally, working speed in the d2-R improved with decreased offset values. Thus, our results suggest that both, slope and offset, are linked with affect, mood, and cognition. Importantly, we found that both aperiodic features were strongly correlated with each other, which is most likely caused by the rotation of the slope (Podvalny et al. 2015) as shown schematically in Fig. 1. Consequently, we cannot interpret slope and offset independently of each other. Nevertheless, our findings support other studies reporting a relationship between the 1/f activity and cognition, for instance, in terms of cognitive speed (Ouyang et al. 2020), reaction times (Immink et al. 2021), and performance in short-term working memory (Thuwal et al. 2021). However, the exact relationship and, in particular, the functional distinction of both aperiodic features remain to be elucidated.

Similar to the slope, the offset was also decreased after both runs, meaning that the cumulative neuronal firing rate was decreased (Miller et al. 2007, 2009; Manning et al. 2009). Higher blood oxygen level dependent (BOLD) signals in fMRI are associated with higher offset values (Winawer et al. 2013; Jacob et al. 2021) implying that brain metabolic processes decreased due to lower neurophysiological activation. Reduced BOLD signals accompanied by improved cognitive performance were also evident in a long-term exercise intervention, indicating more efficient information processing (Voelcker-Rehage et al. 2011). Thus, the reduced offset could neurophysiologically explain the improved working speed of the d2-R we found after exercise. An offset reduction was also demonstrated in an isolation study within the area of space research, where it was hypothesized that several months of sensory deprivation led to this effect (Weber et al. 2020). Therefore, the present data might support the notion that 30 min of running causes higher sensory inhibition leading to a reduced amount of information that requires response and is processed, which is reflected by an offset reduction.

In addition to the aperiodic activity, we found running-induced changes in oscillatory activity. In particular, the eyes open condition showed an increased 1/f-corrected alpha activity 25 min after SR. An increase in alpha activity has initially been linked to a state of decreased cortical activation (Klimesch 1999; Niedermeyer 1999) and has lately been discussed to selectively suppress task-irrelevant sensory input (Foxe and Snyder 2011; Foster and Awh 2019). This active inhibitory gating of information processing (Jensen and Mazaheri 2010; Başar 2012; Peterson and Voytek 2017) affects selective attention (Payne and Sekuler 2014), which might explain the improvements in the cognitive performance which was demonstrated here. Furthermore, alpha activity is functionally associated in the frontal area with positive mood states (Norwood et al. 2019) and with relaxation states (Klimesch 1999; Niedermeyer 1999). However, as SR did not prove to be significantly superior in terms of benefits for mood or cognition, these electrocortical differences between the running conditions were not clearly reflected in brain function. Furthermore, the 1/f-corrected activity in the low beta band was reduced after both runs. Corresponding changes in these frequency bands following exercising have already been found in other studies (Schneider et al. 2009a; Brümmer et al. 2011), even if the overall findings are heterogeneous (Gramkow et al. 2020). However, as the brain activity conflates both, oscillatory and aperiodic activity (Donoghue et al. 2020; Ostlund et al. 2022), what was not taken into account in these studies, it is unclear to what extent true oscillations have shifted. Therefore, we suggest that future studies investigating exercise-induced changes in neural activity distinguish between changes in rhythmic and arrhythmic activity. As outlined, our results support the relevance of both neuronal components, which are important to better understand the underlying neurophysiological mechanisms of psychological improvements achieved by physical activity.

### Limitation

We address three major limitations of the present study. (1) The non-randomized study design contains the risk of order effects. The lack of randomization is explained by the fact that the individual feel-good intensity should be prescribed, which is why we had to determine this intensity first. (2) The perceived autonomy, which is according to SDT considered to be particularly important for exercise-induced effects on psychological outcomes (Edmunds et al. 2008; Ekkekakis 2009; Legault and Inzlicht 2013; Fraguela-Vale et al. 2020) was intentionally not assessed, as it could not be guaranteed that the subjects, even with their background as sport science students, could have otherwise anticipated the aim and theoretical background of the study. (3) Regarding electrocortical activity, it should be noted that between both post-measurements (post5 and post25), the questionnaires for mood were filled out and lactate sampling and cognitive tests were performed. Consequently, a shift in attention cannot be excluded, which may have had an influence on brain activity in the subsequent post25 measurement. However, this does not impact the comparisons between the running conditions, as the same protocol was performed for both interventions.

### Conclusion

In conclusion, we demonstrated that both, self-selected running (SR) and imposed running (IR) led to partially improvements in mood and cognition. Brain activity revealed that the aperiodic (1/f) features decreased after running, meaning that spectral slope was steeper, and the offset was reduced. This shift in cortical excitation towards an enhanced neural inhibition might help to explain the psychological improvements after running. This is supported by the (small) correlations we found between the aperiodic features and mood and cognition. Additionally, we observed an increase in 1/f-corrected alpha activity after SR only and a decrease after both runs in the 1/f-corrected low beta band. These electrocortical differences between the running conditions were not clearly reflected in brain function, as SR did not prove to be significantly superior in terms of benefits for mood or cognition. However, considering on the one hand that the physical workloads of both runs were identical, and on the other hand that we examined experienced athletes who were used to receiving instructions, it remains remarkable that there was any evidence of an influence of external instructions at all. The importance of autonomy during exercising and the impact on mood and cognition requires further investigation, but here, we provided further insights into the underlying mechanisms in the brain.

## Supplementary Information

Below is the link to the electronic supplementary material.

Supplementary file1 (DOCX 596 KB)

## Data Availability

The data of the current study can be retrieved from the corresponding authors at reasonable request. The code for the EEG analysis was based on the FieldTrip Toolbox (Oostenveld et al. 2011) and can be retrieved from the corresponding authors at reasonable request.
