# Supplementary material for: Self-selected versus imposed running intensity and the acute effects on mood, cognition, and (a)periodic brain activity
Source: Cogn Neurodyn. 2024 Mar 1;18(5):2221–41. doi: 10.1007/s11571-024-10084-2 (PMC11564500; doi:10.1007/s11571-024-10084-2)
Supplement: Supplementary file 1 — Supplementary file1 (DOCX 596 KB) [file 11571_2024_10084_MOESM1_ESM.docx]

**Supplementary Information**

**Self-selected vs. imposed running intensity and the acute effects on mood, cognition, and (a)periodic brain activity**

Leonard Braunsmann^1*^, Finja Beermann^2^, Heiko K. Strüder^1^, Vera Abeln^1^

^1^ Institute of Movement and Neurosciences, German Sport University Cologne, Cologne, Germany

^2^ Albert-Ludwigs University of Freiburg, Freiburg, Germany

*Corresponding author

Leonard Braunsmann, Institute of Movement and Neurosciences, German Sport University Cologne, Am Sportpark Muengersdorf 6, 50933 Cologne, Germany.

Email: l.braunsmann@dshs-koeln.de

Phone: +49 221 4982 4261

***Table S1*:** (Repeated) ANOVA with temperature as covariate.

|  | ***F* statistics** | ***p*** | ***partial η²*** |
| --- | --- | --- | --- |
| Feeling Scale | 7.75 | .007* | .126 |
| Felt Arousal Scale | 0.28 | .599 | .005 |
| MoodMeter® PEPS | 0.64 | .133 | .041 |
| MoodMeter® PSYCH | 0.39 | .537 | .007 |
| MoodMeter® MOT | 0.87 | .099 | .050 |
| Digit span forwards | 0.97 | .330 | .018 |
| Digit span backwards | 0.13 | .717 | .002 |
| Digit span overall | 0.86 | .770 | .002 |
| d2-R WA | 2.15 | .148 | .038 |
| d2-R WS | 0.90 | .348 | .016 |
| d2-R CP | 1.58 | .215 | .028 |
| Speed | 0.05 | .825 | .001 |
| Heart rate | 3.30 | .075 | .060 |
| Lactate pre | 0.63 | .433 | .011 |
| Lactate post1 | 4.17 | .064 | .080 |
| Lactate post10 | 3.92 | .053 | .066 |
| Borg RPE | 7.63 | .008* | .124 |
| Impact of COVID-19 | 0.15 | .679 | .003 |

*p* = significance value; *η²* = effect size via eta squared; * = significant (*p* < .05); PEPS = physical state; PSYCH = psychological strain; MOT = motivational state; WA = working accuracy (sum of all errors in relation to WS); WS = working speed (WS; sum of crossed-out targets); CP = concentration performance (number of crossed-out targets minus errors of commission; RPE = rating of perceived exertion.

**Post-hoc analysis**: After adjusting for temperature, Bonferroni-corrected post-hoc analysis revealed a significant difference between SR and IR in the Feeling Scale (FS; *p* = .007, M_Diff_ = -1.03, 95%-CI[-1.56, -0.50]), and the Borg rating of perceived exertion (RPE; *p* = .008, M_Diff_ = 1.89, 95%-CI[0.52, 3.26]).

**Table S2:** Correlations between aperiodic features, slope and offset, and psychological parameters.

| **Eyes Open** | **Slope** | | **Offset** | |
| --- | --- | --- | --- | --- |
|  | ***r*** | ***p*** | ***r*** | ***p*** |
| Feeling Scale | -.094 | .318 | .025 | .793 |
| Felt Arousal Scale | **-.236** | **.011*** | -.080 | .399 |
| MoodMeter® PEPS | .136 | .150 | **-.234** | **.012*** |
| MoodMeter® PSYCH | -.086 | .361 | .023 | .806 |
| MoodMeter® MOT | -.138 | .144 | .006 | .952 |
| Digit span forwards | -.177 | .060 | **.219** | **.019*** |
| Digit span backwards | -.168 | .074 | .137 | .147 |
| Digit span overall | **-.206** | **.028*** | **.211** | **.024*** |
| d2-R WA | **-.215** | **.022*** | .100 | .289 |
| d2-R WS | .162 | .084 | **-.260** | **.005*** |
| d2-R CP | .028 | .770 | -.152 | .107 |
| **Eyes Closed** | **Slope** | | **Offset** | |
|  | ***r*** | ***p*** | ***r*** | ***p*** |
| Feeling Scale | -.025 | .796 | .087 | .360 |
| Felt Arousal Scale | **-.195** | **.037*** | -.036 | .701 |
| MoodMeter® PEPS | .153 | .104 | -.177 | .059 |
| MoodMeter® PSYCH | .003 | .975 | .014 | .881 |
| MoodMeter® MOT | -.014 | .884 | -.024 | .800 |
| Digit span forwards | -.080 | .395 | .173 | .065 |
| Digit span backwards | -.098 | .301 | .104 | .270 |
| Digit span overall | -.110 | .244 | .167 | .075 |
| d2-R WA | **-.211** | **.025*** | .085 | .371 |
| d2-R WS | -.065 | .429 | -.057 | .548 |
| d2-R CP | -.181 | .053 | .042 | .660 |


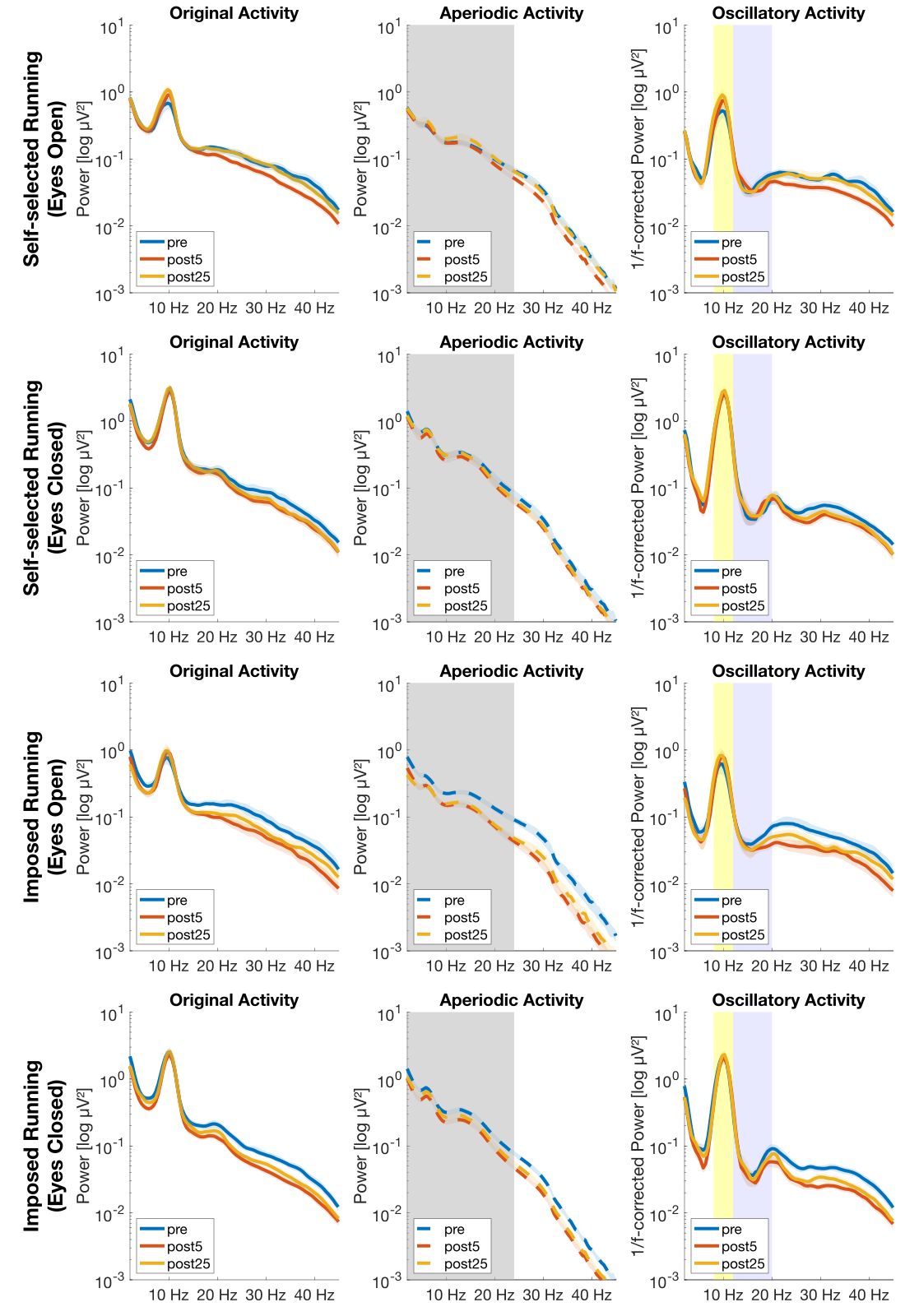


***Figure S1****: Power spectral density (PSD) plots showing how the original EEG data was disentangled into the aperiodic and oscillatory components. PSD plots are presented as mean ± SEM in semi-log power space.* ***(Left)*** *PSD consisting of both, the aperiodic and oscillatory components.* ***(Middle)*** *PSD after IRASA. The gray area marks the frequency range in which the aperiodic features were calculated (~1.9 to 23.7 Hz).* ***(Right)*** *PSD in which the aperiodic activity (1/f) was subtracted for the pure oscillations. The yellow area marks the alpha band (8-12 Hz), and the violet area marks the low beta band (12-20 Hz).*

**
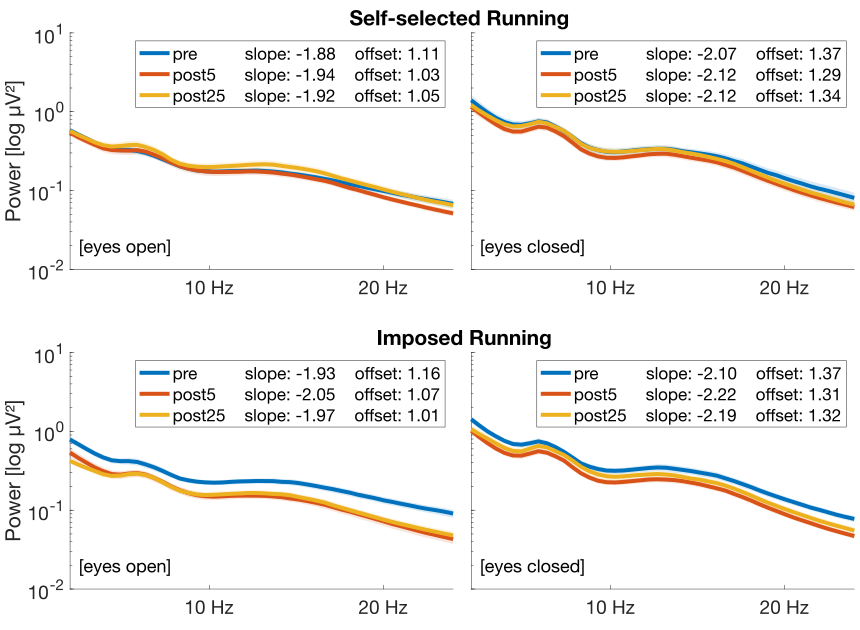
*Figure S2:*** *Grand average aperiodic power spectral density (PSD) after IRASA. Grand averages were calculated across all participants and channels. PSD plots are presented as mean ± SEM in semi-log power space.*
